# A qualitative analysis of Medicaid beneficiaries perceptions of prenatal and immediate postpartum contraception counseling

**DOI:** 10.1177/17455057221124079

**Published:** 2022-09-15

**Authors:** Lindsey Yates, Sarah Birken, Terri-Ann Thompson, Gretchen S Stuart, Sandra Greene, Kristen Hassmiller Lich, Morris Weinberger

**Affiliations:** 1 Department of Maternal and Child Health, Gillings School of Global Public Health, University of North Carolina at Chapel Hill, Chapel Hill, NC, USA; 2 Department of Implementation Science, Bowman Gray Center for Medical Education, Wake Forest School of Medicine, Winston-Salem, NC, USA; 3 Ibis Reproductive Health, Cambridge, MA, USA; 4 Department of Obstetrics and Gynecology, UNC School of Medicine, University of North Carolina at Chapel Hill, Chapel Hill, NC, USA; 5 Department of Health Policy and Management, Gillings School of Global Public Health, University of North Carolina at Chapel Hill, Chapel Hill, NC, USA

**Keywords:** contraception counseling, maternal health, Medicaid, postpartum contraception, racial disparities

## Abstract

**Objectives:**

In the United States, about four out of every ten births are financed by Medicaid, making it a program that is key in addressing racial disparities in maternal health. Many women covered by Medicaid have access to prenatal and immediate postpartum contraception counseling that can aid them in their postpartum contraception decision-making. However, existing inequities within Medicaid and a history of reproductive harms targeting Black women and women with low incomes may contribute to women with Medicaid having different experiences of contraception counseling. This qualitative study explores how Black women and White women insured by Medicaid perceive prenatal and immediate postpartum contraception counseling and identifies additional factors that shape their contraception decision-making.

**Methods:**

We conducted semi-structured interviews with 15 Medicaid beneficiaries who delivered at a public teaching hospital in North Carolina. Interviews focused on women’s beliefs about planning for pregnancy, experiences with prenatal and immediate postpartum contraception counseling, and perceived need for postpartum contraception. We used a priori and emergent codes to analyze interviews.

**Results:**

Seven Black women and eight White women completed interviews 14–60 days postpartum. All women reported receiving prenatal and immediate postpartum counseling. Several women described receiving prenatal counseling, reflective of patient-centered contraception counseling, that helped in their postpartum contraception decision-making; one woman described receiving immediate postpartum counseling that helped in her decision-making. Some Black women reported receiving unsupportive/coercive contraception counseling. In addition to contraception counseling, past reproductive health experiences and future pregnancy intentions were salient to women’s contraception decision-making.

**Conclusions:**

Prenatal and immediate postpartum contraception counseling can help some Medicaid beneficiaries with their postpartum contraception decision, but past reproductive health experiences and future pregnancy intentions are also relevant. Counseling that does not consider these experiences may be harmful, particularly to Black women, further contributing to racial disparities in maternal postpartum health outcomes.

## Introduction

Approximately 42% of all births in the United States are financed by Medicaid.^1^ Given the size and scope of the program, Medicaid plays an important role in addressing racial disparities in maternal health outcomes, including ensuring access to postpartum contraception.^2–4^ Providers are encouraged to discuss options for postpartum contraception with all women during pregnancy, and again after delivery to help them reach their reproductive goals.^5^ The American Academy of Pediatrics and the American College of Obstetricians and Gynecologists recommend providing contraception counseling to all women during their prenatal visits and immediately postpartum.^5^ One multi-state sample found that postpartum contraception use was highest among women who received both prenatal and immediate postpartum contraception counseling.^6^ For women with low incomes, contraception counseling during the prenatal and immediate postpartum periods is key. Women who qualify for Medicaid because of pregnancy may lose access to helpful contraception services within weeks after delivery.^7^ Although there are increased state efforts to extend postpartum Medicaid coverage,^8^ inequities within the Medicaid program persist^9^ and may negatively impact some women’s postpartum contraception decision-making.^10,11^

Racial inequities within Medicaid are the result of structural racism which is the “normalization and legitimization of an array of dynamics—historical, cultural, institutional and interpersonal—that routinely advantage whites while producing cumulative and chronic adverse outcomes for people of color.”^12^ These inequities are evident in the racial disparities among Medicaid beneficiaries. For example, Black women are twice as likely to have their births covered by Medicaid, compared to White women.^13^ Black women’s overrepresentation in the Medicaid population is the result of structural inequities that contribute to Black women having lower incomes.^14^ Inequities in Medicaid are further exacerbated by obstetric racism. Obstetric racism, systematic racism effecting the ways in which women are treated during conception, pregnancy, labor and delivery, and postpartum,^15,16^ harms contraception counseling discussions that take place between providers and women. For example, prior research has shown that Black and Latina women with low incomes are more likely to report being told to limit their childbearing compared to middle-class White women.^17^ Other studies have found that providers are more likely to recommend permanent contraception or long-acting reversible contraception (LARC) to Black women and women with low incomes.^18,19^ In addition, there is a history of reproductive coercion (including the forced sterilization of poor, Black, Latino, and developmentally disabled individuals) and the targeted distribution of some contraception methods to poor Black communities.^20^ For some women, this history deepens their mistrust for contraception and contraceptive services.^21^

Prenatal and immediate postpartum contraception counseling can offer important benefits and may be particularly useful to women with Medicaid, but because of inequities within the Medicaid program and a history of reproductive harms targeting Black women and women with low incomes, it is important to assess the contraception counseling experiences of women insured by Medicaid. This article examines Black women’s and White women’s, insured by Medicaid, decisions about postpartum contraception, including their perceptions of contraception counseling. We also describe the beliefs, knowledge, and reproductive health needs that influence postpartum contraception decision-making.

## Methods

### Procedures

We conducted semi-structured interviews with women who had live, singleton, full-term births at a public teaching hospital in North Carolina between December 2019 and October 2020. Participants were Medicaid beneficiaries, aged 18 years or older who identified as non-Hispanic Black or non-Hispanic White and spoke English. Women were excluded if they experienced a complicated birth, with a serious adverse event affecting the mother or infant. Women were recruited through (1) inpatient recruitment and (2) a website that provided information about research opportunities available to the public. From December 2019 to February 2020 the first author (L.Y.) reviewed the daily inpatient census and approached all eligible women during their delivery hospitalization about the study. From May 2020 to December 2020 the website listed relevant study information. Women who contacted study staff through the study website were screened to determine their eligibility. The first author (L.Y.) contacted all women who expressed interest in the study by phone, email, or text message approximately 14 days after delivery to schedule telephone interviews. The first author (L.Y.) conducted all interviews with participants.

All eligible and interested participants received a written copy of the consent. All participants completed the verbal informed consent process before the recorded interview as part of the interview procedures. All participants were sent a US$25 gift card after the interview. All study procedures were approved and exempted by the University of North Carolina Institutional Review Board (study no.: 19-0798).

### Measures

**Figure 1.**
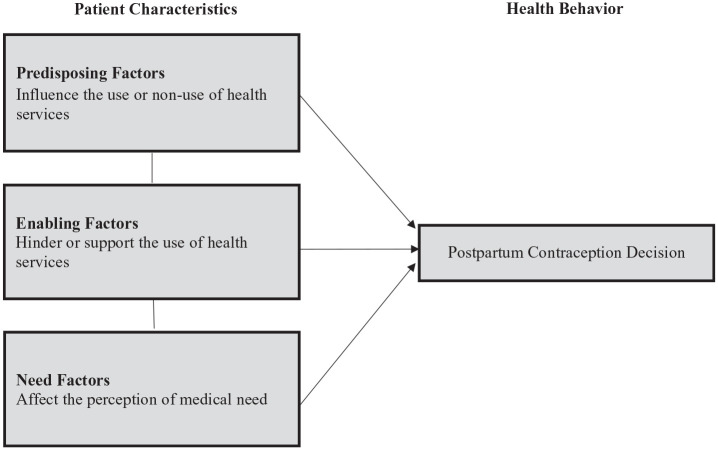
Behavioral Model of Health Services Use adapted to explore postpartum contraception decision-making.

Interview questions were derived from the Behavioral Model of Health Services Use (BMHS) (Figure 1).^22^ This model posits that various factors influence an individuals’ decision to seek or receive care for a health condition. We used this model to examine postpartum contraception decision-making. Factors highlighted by the model include predisposing factors such as beliefs about planning for pregnancy, enabling factors such as contraception counseling received during the prenatal and immediate postpartum periods, and need factors such as perceived need for postpartum contraception.^23^ The interview questions explored several topics including beliefs about contraception, use of contraception, choice of contraception after delivery, sources and resources used to learn about contraception, experiences with prenatal and immediate postpartum care and contraception counseling, and perceptions about short-interval pregnancies. Interview questions were piloted before participants were enrolled. Information collected from the pilot interviews were used to improve the interview format and not included in the final analysis.

### Data analysis

The interviews were recorded and transcribed verbatim and analyzed by two study team members, one Black woman (L.Y.) and one White woman (B.W.), using template analysis. The coding template was based on the BMHS. Both team members used MAXQDA, version 12 (VERBI Software GmbH, Berlin), to code each transcript independently. The team members reviewed each transcript together to reach consensus on themes and applied the appropriate codes for each interview. As new themes emerged, team members re-reviewed interviews using the updated coding template. We reached data saturation when we could not identify any additional codes.

A copy of the Consolidated Criteria for Reporting Qualitative Research (COREQ) checklist and the interview guide in supplementary materials.

## Results

We completed interviews with seven Black and eight White women 14 to 60 days postpartum. The participants ranged in age (20–38 years) and had various pregnancy histories (1–10 pregnancies; Table 1). Women reported receiving prenatal care from teaching hospitals, local health departments, or other medical practices. Four women reported transferring care from one practice to another practice for their prenatal care. Thirteen women reported that they attended all or most of their prenatal visits.

**Table 1.** Characteristics of participants.

	Black (n = 7)	White (n = 8)
Age (years)		
20–24	4	2
25–29	–	3
30–34	–	3
35–40	3	–
Number of previous total pregnancies		
1–3	5	4
4 or more	2	4
Prenatal care location		
Local health department	2	3
Teaching hospital clinic	2	2
Other medical practice	3	3
Frequency of prenatal care		
Attended all/most visits	6	7
Attended some/few visits	1	1
Type of contraception		
Bilateral tubal ligation/Mirena IUD	3	5
Pills/condoms	2	2
Undecided	1	1
Unreported	1	-

IUD: intrauterine device.

Twelve of the 15 women reported selecting a contraceptive method by the time of their interview. Three Black and two White women selected bilateral tubal ligation. Three white women selected LARC that they planned to receive at their postpartum visit. Two women, one Black and one white, were considering LARC, but had not made a final decision. None of the five women who selected or considered LARC reported receiving prenatal counseling about the option to receive a LARC method after delivery, but before discharge.

A summary of findings is available in Table 2. We describe five themes among the predisposing, enabling, and need factors of the BMHS. When appropriate, we note differences within themes among the Black respondents and White respondents. We present quotes with the pseudonyms (selected by the first author) and age of the participants.

**Table 2.** Participants’ perceptions of factors influencing postpartum contraception decision-making.

Factors and Themes	Summary of findings
Predisposing Factors	
Theme 1. Beliefs, knowledge, and other sources of information shape perceptions of contraception	Women’s perceptions of contraception were influenced by desire for planned pregnancies, direct or indirect experience with short-interval pregnancies, and information about contraception from friends, family and social media.
Enabling Factors	
Theme 2. Prenatal counseling can help or hinder contraception decision-making	All women received one of three types of prenatal counseling: (1) active, ongoing counseling, (2) limited but satisfactory counseling, and (3) unsupportive, coercive counseling. Four White women and two Black women described their prenatal contraception counseling as helpful in their decision-making. Three Black women described receiving unsupportive, coercive counseling.
Theme 3. Immediate postpartum counseling has limited effects on contraception decision-making	All women received postpartum contraception counseling during their delivery hospitalization. Generally, these discussions focused on confirming the contraception decision made during prenatal care. One White woman found these discussions helpful in her decision-making. The three Black women who reported unsupportive, coercive prenatal counseling reported continuing to feel pressured about their contraception choice after delivery.
Theme 4. Medicaid coverage influences access to contraception and prenatal care	The low/no-cost of contraception under Medicaid was relevant to some women’s decision-making and many women were able to easily access Medicaid. For a few the 60-day limit on postpartum coverage influenced their contraception decision, and for one woman Medicaid approval came later in her pregnancy impacting her access to prenatal care.
Need Factors	
Theme 5. Physiological effects of contraception and pregnancy impact decisions about contraception	All women reported prior contraception use. Negative health effects resulting from the use prescription contraception (e.g. weight gain, pain or discomfort, mood swings, irregular bleeding), influenced their selection of postpartum contraception. Their plans and desire for more children were also salient to their decision-making.

## Predisposing factors

### Theme 1. Beliefs, knowledge, and other sources of information shape perceptions of contraception

#### Beliefs about planning for pregnancy

There were no notable racial differences among participants about their beliefs. All women described planned pregnancies as more desirable than unplanned pregnancies. However, they acknowledged that planning for pregnancy has challenges, especially if they experience difficulties getting pregnant. They also reported stable income, family leave, and emotional support from partners, friends, and family as resources that women should assess when planning for pregnancy.

#### Knowledge and experience with short-interval pregnancy

All participants reported that either they or someone they knew became pregnant shortly after giving birth. Several women specifically stated that pregnancy within “six weeks” or before the “six-week postpartum visit” was possible. Most women viewed a short-interval pregnancy as undesirable and reported planning to use contraception after delivery to prevent this. A few women with recent short-interval pregnancies noted that their current pregnancy after the short-interval pregnancy was a reason for their desire to use a long-acting method.

#### Information from other sources

All but one participant reported receiving information or feedback about specific types of contraception from friends and family, online searches, and/or social media. A few women reported trusting the information they received from other sources more than information they received from their provider. Multiple women reported that discussions with family were encouraging; one Black woman and one White woman found advice from family members was less helpful because it did not consider their specific needs. For example, Ashely, a 28-year-old White woman described feeling frustrated by her mother’s suggestions to get bilateral tubal ligation:

It aggravated me because . . . she was like, “You don’t need no more kids, you don’t need any more kids, you don’t need any more kids” and when I had my third baby . . . she was just pressuring me to do it . . . (Ashely, 28 years)

## Enabling factors

### Theme 2. Prenatal counseling can help or hinder contraception decision-making

During their prenatal care, all participants received one of three categories of contraception counseling: (1) active, ongoing; (2) infrequent, but satisfactory; or (3) unsupportive/coercive.

#### Active, ongoing counseling

Eight women reported having conversations about various contraception options during most or all of their prenatal visits. Some women described that these conversations included a discussion of prior contraception use. Most women noted that the provider was clear that they had the autonomy to make a different contraception choice at any time. Six women, two Black and four White, described these discussions as helpful. Typically, these women were deciding between two contraceptive options or were unsure of their contraception choice. Samantha, a 30-year-old White woman, characterized this type of counseling:

We talked about tying my tubes. They talked to me about the different birth control [because] I can only take certain birth control because I have a history of [PCOS]. So, they helped me to figure out what I could do in order to keep, you know, my symptoms down and to control those as well. So, we talked about what birth controls I can take that would be safe for me . . . Pretty much every month we talked about it . . . At first, it was like once a month, and then when I was going every two weeks or so, as I got further along . . . um, you know, the conversation still came up about what I was thinking about and what I was going to go with. (Samantha, 30 years)

#### Infrequent, but satisfactory counseling

Four women, one Black and three White, reported having three or fewer conversations about contraception during their pregnancy. All four women were very sure of their contraceptive choice (three selected a long-acting method and one selected pills) and felt satisfied with the information their providers offered. Prenatal providers using this style typically did not describe more than one contraceptive method. Two women sought additional information from other sources to make their final contraception decision. Hannah’s (White, aged 21 years) experience is typical:

L.Y.: When you were going, did you all talk about birth control after having the baby?

Hannah: No. Well, yes, yes, yes, they did ask me what I wanted to do after. I said I’m going to go on the pill.

L.Y.: How many times did they ask you about it?

Hannah: I think it was once or twice . . . there, towards the end of my pregnancy. They were just asking me if I had thought about any birth control afterwards.

L.Y.: Okay. Did they mention any other types of birth control other than the pill?

Hannah: I don’t think so. No.

L.Y.: Okay. Overall, what did you think about like those conversations that you had about birth control with the doctors and nurses there?

Hannah: They were okay I guess, since I already knew what I wanted to go on and I didn’t really like, go into detail and ask myself about anything. So, I think it was okay.

#### Unsupportive/coercive counseling

Three young Black women said that their contraception choice or concerns were dismissed or minimized by the prenatal provider. Two women felt pressured to get an intrauterine device (IUD), while one woman was discouraged from selecting her desired choice of permanent contraception. The three women reporting this type of counseling described how they felt during these conversations with their providers:

It was more so like Mirena, Mirena, Mirena. This is the one. I’m like, oh, my God . . . And it’s like ok. It scares me because I’m like, why [do] you want me to get that so bad? What is it? It makes me nervous . . . I feel like that’s one of the ones that’s being pressured . . . It was always they want me to get the Mirena and how good the Mirena is, how good it lasts, and how efficient it is. That’s all I heard. . . . That’s why I said it makes you feel like you’re being pressured, like what are you all getting out of getting me on this. Like what in the world! . . . I don’t know. That’s why I decided, so I don’t feel like I’m being targeted, how [about] I’m just going to use condoms then. Then I don’t have to worry about if they just put this on me or want me to take it so bad for a reason. (Destiny, 23 years)

Oh yes! It’s obvious, there’s a huge difference in minorities of how they push birth control on us. Because I’m telling you, every doctor asked me, “What is your birth control plan?” Control what birth! . . . I wouldn’t be too shocked if they pushed birth control on more minorities. And especially high-risk, and with me [having] sickle cell, they’re trying to push it on me because, I don’t know why . . . they don’t want to . . . deal with the high-risk of the pregnancy, maybe it costs too much, who knows . . . (Jackie, 20 years)

I actually wanted to get my tubes tied, but everyone talked me out of doing that one . . . They didn’t say I couldn’t. They were just telling me that they wouldn’t recommend it because I was still so young . . . I don’t want to say [they] talked me out of it, but I don’t think that she [wrote]that down. I think she wanted me to decide again. She kind of like wanted me to think about it some more. (Angel, 21 years)

These women wanted conversations that were more considerate of their choices and discussed more comprehensive information about their contraception options.

### Theme 3. Immediate postpartum counseling has less effect on contraception decision-making

Generally, participants who did not receive permanent contraception reported that the postpartum contraception counseling they received before discharge focused on confirming the method they selected during prenatal care. A small number of women reported slightly different experiences with postpartum contraception counseling during the immediate postpartum period. In one case, a White woman reported her postpartum provider suggested a different method than her prenatal provider because of potential contraindications with the previously recommended method. Two Black women who desired a bilateral tubal ligation immediately following delivery had their procedures rescheduled due to complications they experienced. A White woman who was undecided but considering an implant was offered immediate postpartum implant placement but declined.

The Black women who reported unsupportive/coercive prenatal counseling reported continuing to feel pressured about their contraception choice after delivery. Jackie (a 20-year-old Black woman) and Angel (a 21-year-old Black woman) ultimately selected contraception inconsistent with their preference to avoid further discussion:

. . . They were on me in the hospital like, “Birth control! Birth control!” I was just like, Oh my god, I just had a baby. I don’t know if I want to be on birth control because I’m trying to heal from a scar . . . Yeah, that’s when I said it again. I said, “I think I am just going to get the IUD,” because they asked me when I got there, when I was in labor, and they asked me after labor. (Jackie, 20 years)

They asked me again and I told the lady again, and then she tried to talk to me out of it also, so then that’s when I just decided on the pills. Because I had already forgot about all the other choices. I was just, “I’ll just do the pills.” Because you know, I just had a baby I was still drugged up and everything so I was like I’ll just do the pills. (Angel, 21 years)

Two women described challenges related to contraception uptake and postpartum care after discharge. Jackie, a 20-year-old Black woman, contacted her postpartum provider after discharge and felt dismissed when she told the provider she was not getting contraception:

I was supposed to go to the doctor, you know, right after you give birth you go to the doctor because I had a C-section—but I feel like the fact that I wasn’t getting birth control is the reason they pushed my appointment so far back. (Jackie, 20 years)

Amber, a 27-year-old White woman, said she was not given adequate information about when or how to take the pills. When she called her postpartum provider for help, she was told she needed to attend her postpartum appointment to receive more information.

### Theme 4. Medicaid coverage influences access to contraception and prenatal

Having Medicaid also influenced women’s choice of contraception. Multiple women, specifically noted that Medicaid covered the cost of their chosen contraceptive method, including a few White women who described choosing bilateral tubal ligation because Medicaid covered the procedure costs. However, a few women experienced coverage challenges with Medicaid. For example, two women knew that Medicaid for Pregnant Women ends 60-days postpartum, which impacted their contraception decision-making. Crystal, a 35-year-old Black woman, who was deciding between bilateral tubal ligation and an IUD said,

I have the family Medicaid so it’s totally different now. I mean they will keep my regular prenatal Medicaid until my 6-week checkup and then it’s going to cut off and then it will be family Medicaid . . . family Medicaid only covers certain stuff. Like the family Medicaid is not going to cover me getting my tubes tied but pregnancy Medicaid will cover me getting my tubes tied because it is considered contraceptives for you know pregnancy . . . But it’s not considered that with family Medicaid. Family Medicaid is only going to do my IUD it will not do the [tubal ligation]. (Crystal, 35 years)

Emily, a 24-year-old White woman, was concerned that if she had issues with a selected contraception method, she would be unable to change it after her Medicaid coverage ended:

I wasn’t really too big on taking that risk of having the same problems over again, and then me having to go through another surgery in the future. Because that’s something that I was eventually going to have to get done if the birth control didn’t work . . . Which is harder to get our insurance to pay for, too . . . And I don’t have Medicaid. I did have pregnancy Medicaid, so the thing with that is once you’ve surpassed your six to eight-week postpartum period, what do you do after that? So even though you can still get birth control options for free, if I decided to have a [tubal ligation] after that point, it would be like, okay, well, how am I going to pay for this? That’s an invasive surgery, and at least a one-night hospital stay, so ten grand at the cheapest. That’s a lot of money for the average person to come up with . . . (Emily, 24 years)

Several women noted that applying for Medicaid was simple, and their local health departments were instrumental in their application and approval process. One Black woman and one White woman reported that their Medicaid approval came late in their pregnancies. For the Black woman that delay contributed to her beginning prenatal care later in pregnancy.

## Need factors

### Theme 5. Physiological effects of contraception and pregnancy impact decisions about contraception

All participants reported prior contraception use. Their postpartum contraception preference was primarily shaped by previous experiences including side-effects (e.g. weight gain, pain or discomfort, mood swings, irregular bleeding). Women who selected the Mirena IUD postpartum had very positive prior experiences, including not having a period. Two of the three women who choose pills and two who were undecided, chose a different postpartum contraceptive because of prior negative experiences with previous methods.

Participants’ future pregnancy intentions were shaped by their pregnancy experiences. About half of the participants indicated that their recent pregnancy was planned, with no distinct racial differences among women who described their pregnancy as planned. All the women who received a bilateral tubal ligation and two of the women who selected the Mirena IUD were clear that they did not want to be pregnant again. While a few women attributed this decision to severe physical health challenges during pregnancy; others stated their age and spacing of their children as a reason they did not want to be pregnant again. The remaining women did not state their future pregnancy intentions or were open to a future pregnancy.

## Discussion

Our findings suggest that there are racial differences within the Medicaid program affecting Black women’s and White women’s experiences with prenatal and immediate postpartum contraception counseling. Postpartum contraception counseling can help some Medicaid beneficiaries choose methods consistent with their reproductive goals; however, counseling that fails to consider women’s reproductive goals may be less helpful and potentially harmful, especially for Black women.

Although all women in this study received prenatal counseling, only half received active, ongoing prenatal contraception counseling. Patient-centered contraception counseling has emerged as a key strategy to help all women achieve their reproductive goals.^24,25^ Patient-centered contraception counseling involves building trust through an interpersonal relationship; eliciting and appropriately responding to patients’ preferences; providing accurate information about utilization and side-effects; helping patients make contraception decisions based on their preferences and options available to them; and recognizing root causes of disparities.^24,26,25^ Consistent with previous literature,^24,26,27^ counseling reflecting a patient-centered approach that included elements of shared decision-making, prioritized women’s preferences, provided accurate information, and offered guidance was especially useful to women considering multiple conception options.

Of note, three Black women reported receiving unsupportive/coercive counseling and feeling forced to make contraceptive choices they did not want. There is growing evidence that Black women feel pressured to make certain contraceptive choices, including using LARC^17,28,29^ and that providers, in fact, make different recommendations for contraception based on patients’ race.^19^ This type of dismissive and coercive treatment is reflective of obstetric racism.^15,16^ Unsupportive/coercive counseling is inconsistent with patient-centered counseling, as well as reproductive justice.^30^ Reproductive justice recognizes the right to bodily autonomy, deciding when and if to have children, and parent children in ways that are reflective of individuals’ goals and values.^31^ Postpartum contraception counseling should be grounded in the reproductive justice framework and acknowledge the systems and history of injustices that contribute to disparities.^25,32^ Coercive practices erode patients’ trust in providers and hinder equitable reproductive health outcomes.

Although there is evidence that immediate postpartum contraception counseling is associated with postpartum contraception use,^6^ our findings suggest discussions during this period have less impact on most women’s postpartum contraception decision-making. This is consistent with previous research showing that contraception counseling before discharge was brief and “unhelpful.”^33^ The immediate postpartum setting may be suboptimal for contraception counseling because providers and new parents are focused on other postpartum issues.^33^ Conversely, immediate postpartum counseling may be beneficial to women who desire LARC.^34^ Although only one woman who was considering an LARC was advised about immediate postpartum LARC in this study, prior research finds women who are considering immediate postpartum LARC prefer frequent, quality prenatal and immediate postpartum discussions, where their autonomy and valid information are prioritized.^35,36^ Counseling women about immediate postpartum LARC during their inpatient postpartum stay is important and should be coupled with prenatal counseling.

In our study, we found that women’s prior contraception use and future pregnancy intentions were relevant to their postpartum contraception decision-making. This is similar to previous work suggesting that contraceptive choice and use are associated with both pregnancy and method-related experiences (e.g. prior unintended pregnancies, dislike of other contraceptive methods, future pregnancy intentions).^37^ The women also believed that planned pregnancies were more desirable than unplanned pregnancies. Unlike some prior research suggesting that women with low incomes may have less clear pregnancy intentions,^38^ half the women reported planning their recent pregnancy, and many described clear future pregnancy intentions.

Consistent with prior literature,^6,39^ about half of the women reported that the cost of contraception under Medicaid contributed to their choice of contraception. Limits on Medicaid coverage for pregnant women diminish women’s access to comprehensive postpartum contraception counseling and options.^7,40^ In addition, women relied on other sources of information, including family and friends, for contraception information. This aligns with research that suggests that social networks influence women’s postpartum contraception decisions.^41^ There is also evidence that among women with low incomes, their desire for contraception is influenced by their partners and families.^42^ Acknowledging these sources of information may be important to women and help them feel more confident with their postpartum contraception decision.

In this study, we explore women’s experiences with contraception decision-making, before they lose their Medicaid coverage. This represents a unique time frame to collect information about perceptions of contraception counseling and reproductive health care. We also used an existing conceptual model to better understand the factors that influence women’s postpartum contraception decisions. This model allowed us to explore various factors, better illuminating the influence of those factors on contraception selection. Despite these strengths, this study has some limitations. First, participants were non-Hispanic Black and non-Hispanic White women and may not reflect the experiences of women from other racial/ethnic groups. Although Black women and White women make up more than 83% of all Medicaid births in North Carolina,^43^ future research should explore the experiences of other historically marginalized populations. Our participants also had uncomplicated deliveries and healthy infants which may have also influenced their experiences with contraception counseling. Second, the study was conducted in one academic medical center in North Carolina. Given our study sample we may have limited the factors influencing postpartum contraception we were able to uncover or the role of hospital environments on these outcomes. Finally, we did not account for provider characteristics (e.g. race, years of licensing, type, location). Future research should explore how such characteristics may influence prenatal and immediate postpartum contraception counseling.

## Conclusion

In our study, although women insured by Medicaid are receiving prenatal and immediate postpartum contraception counseling, many are not receiving patient-centered contraception counseling. Notably, some Black women received counseling that is unsupportive and/or coercive, which could lead to women failing to achieve their reproductive goals and worsen racial inequities in maternal health outcomes within the Medicaid program.

These findings are important for state Medicaid agencies and other stakeholders invested in developing equity-focused solutions to address maternal health disparities in Medicaid. Expanded Medicaid coverage beyond 60-days postpartum is one, fundamental solution to resolve disparities. Additional efforts should focus on addressing obstetric racism, including creating structures that incentivize providers to deliver patient-centered counseling and data measures that assess the quality of contraception counseling for women with Medicaid. Providers and facilities responsible for delivering postpartum contraception counseling should practice counseling is grounded in the reproductive justice framework and prioritizes patients’ individual previous experiences and pregnancy intentions. Developing these types of solutions requires Medicaid agencies to meaningfully engage women with lived experience to help inform programmatic changes to Medicaid. It is also important to acknowledge and understand the policies that limit women with low incomes access to contraception as well as how the changing landscape of women’s access to abortion care may affect women’s decisions about contraception. Addressing inequities in prenatal and immediate postpartum contraception counseling can help reduce racial disparities in maternal health outcomes.

## Supplemental Material

sj-docx-1-whe-10.1177_17455057221124079 – Supplemental material for A qualitative analysis of Medicaid beneficiaries perceptions of prenatal and immediate postpartum contraception counseling Click here for additional data file. Supplemental material, sj-docx-1-whe-10.1177_17455057221124079 for A qualitative analysis of Medicaid beneficiaries perceptions of prenatal and immediate postpartum contraception counseling by Lindsey Yates, Sarah Birken, Terri-Ann Thompson, Gretchen S Stuart, Sandra Greene, Kristen Hassmiller Lich and Morris Weinberger in Women’s Health

sj-docx-2-whe-10.1177_17455057221124079 – Supplemental material for A qualitative analysis of Medicaid beneficiaries perceptions of prenatal and immediate postpartum contraception counseling Click here for additional data file. Supplemental material, sj-docx-2-whe-10.1177_17455057221124079 for A qualitative analysis of Medicaid beneficiaries perceptions of prenatal and immediate postpartum contraception counseling by Lindsey Yates, Sarah Birken, Terri-Ann Thompson, Gretchen S Stuart, Sandra Greene, Kristen Hassmiller Lich and Morris Weinberger in Women’s Health

## Declarations

**Ethics approval and consent to participate:** This study was approved and exempted by the University of North Carolina at Chapel Hill Institutional Review Board (study no.: 19-0798). All eligible and interested participants received a written copy of the consent. All participants completed the verbal informed consent process before the interview that was recorded as part of the interview procedures.

**Consent for publication:** The written and verbal consent included consent to publish. All non-essential identifying details have been omitted.

**Availability of data and material:** The interview guide used to conduct the semi-structured interviews is available in the supplementary materials. Transcripts, recordings, and field notes used for data analysis are available upon request to the author and approval from an Institutional or Ethical Review Board.
