# Supplementary material for: A qualitative analysis of Medicaid beneficiaries perceptions of prenatal and immediate postpartum contraception counseling
Source: Womens Health (Lond). 2022 Sep 15;18:17455057221124079. doi: 10.1177/17455057221124079 (PMC9486062; doi:10.1177/17455057221124079)
Supplement: sj-docx-1-whe-10.1177_17455057221124079 – Supplemental material for A qualitative analysis of Medicaid beneficiaries perceptions of prenatal and immediate postpartum contraception counseling [file sj-docx-1-whe-10.1177_17455057221124079.docx]

| Consolidated criteria for reporting qualitative research (COREQ) Checklist | |
| --- | --- |
| 1. Interviewer/facilitator | Lindsey Yates |
| 2. Credentials | At the time of the interviews the interviewer/PI had her MPH. |
| 3. Occupation | At the time of the interviews the interviewer/PI was a PhD student. |
| 4. Gender | The PI is Female |
| 5. Experience and training relationship with participants | The interviewer/PI had experience recruiting and consenting patients in a hospital setting, as well as experience conducing qualitative interviews with various stakeholders. |
| 6. Relationship established | The PI had no previous relationship with participants prior to beginning the research study. |
| 7. Participant knowledge of the interviewer | The participants were verbally consented by the PI. They were told she was interested in learning about women’s postpartum experiences, specifically what women think about planning for pregnancy and birth control. Whatever that decision may be. |
| 8. Interviewer characteristics | When introducing herself to participants, PI stated that she was a researcher, and mother.  Although she did not explicitly describe herself as a Black woman, but participants may have deduced that information about her when the met or talked with her. |
| 9. Methodological orientation and theory | The PI used template analysis as the methodological orientation. The conceptual model for this study is based on the Behavioral Model of Health Services Use. |
| 10. Sampling | Participants were selected based on a set of inclusion and exclusion criteria. Eligible participants included: women who had live, singleton, full-term births at a public teaching hospital in North Carolina. They were insured by Medicaid, age 18 years or older, identified as non-Hispanic Black or non-Hispanic White, and spoke English. Women were excluded if they experienced a complicated birth, with a serious adverse event affecting the mother or infant. |
| 11. Method of approach | Eligible women were identified by 1) inpatient recruitment and (2) a website that provided information about research opportunities available to the public. |
| 12 Sample Size | Fifteen women participated in the study. |
| 13. Non-participation | Fifteen additional women, who were approached in the hospital expressed interest in participating in the research study, but then later declined or did not respond to follow-up requests to schedule their interview. An additional seven women contacted the PI through the study website, but they did not meet the study inclusion criteria or did not respond to follow-up emails. |
| 14. Setting of data collection | Interviews were conducted by phone. |
| 15. Presence of non-participants | During most interviews the PI was the only person present. However, because some of these interviews took place during the emergency stay at home order issued by the NC governor in response to the COVID-19 pandemic, some interviews took place from the PIs home were here young child was present. Likewise, some participants completed interviews in settings where other family members were present. |
| 16. Description of sample data collection | Seven non-Hispanic Black and eight non-Hispanic White women, insured by Medicaid completed interviews between January 2020 and December 2020. |
| 17. Interview guide | Semi-structured interviews were conducted based on an interview guide. The interview guide was piloted multiple times before the study began. Pilot data was not included in the data analysis. |
| 18. Repeat interviews | No repeat interviews were conducted. |
| 19. Audio/visual recording | Audio recording was used to record interviews. |
| 20. Field notes | Field notes were made during and after interviews. |
| 21. Duration | Interviews lasted approximately 45 minutes. |
| 22. Data Saturation | We reached data saturation when we could not identify any additional codes. |
| 23. Transcripts returned | Transcripts were not returned to participants. |
| 24. Number of data coders | Two coders coded the data. |
| 25. Description of the coding tree | The initial coding template was based on the Behavior Model of Health Services Use. |
| 26. Derivation of themes | Using template analysis, we used of a coding template, developed a priori, that emphasizes a hierarchical structure of broad themes which become progressively narrow. As data are analyzed, the coding template was modified to include emerging, or remove previously-included, themes. The final template was used to interpret findings. |
| 27. Software | We used MAXQDA, version 12 (**VERBI Software GmbH,** Berlin) |
| 28. Participant checking reporting | Participants did not provide feedback on the findings |
| 29. Quotations presented | Quotations were presented with participant pseudonyms (selected by the PI), to illustrate themes and key findings. Various participants quotations are included. |
| 30.Data and findings consistent | We summarize key findings and include quotations, to highlight major and minor themes. |
| 31. Clarity of major themes | Major themes are listed in the paper and in Table 2. |
| 32. Clarity of minor themes | There is a description of diverse cases within relevant themes. |
