# Supplementary material for: A qualitative analysis of Medicaid beneficiaries perceptions of prenatal and immediate postpartum contraception counseling
Source: Womens Health (Lond). 2022 Sep 15;18:17455057221124079. doi: 10.1177/17455057221124079 (PMC9486062; doi:10.1177/17455057221124079)
Supplement: sj-docx-2-whe-10.1177_17455057221124079 – Supplemental material for A qualitative analysis of Medicaid beneficiaries perceptions of prenatal and immediate postpartum contraception counseling [file sj-docx-2-whe-10.1177_17455057221124079.docx]

**Introduction – paragraph of intent**

Hi Ms (Patient Name)

This is Lindsey Yates. We met at UNC. I’m calling about the interview we scheduled. Is now still a good time?

(If YES) – Continue with the script

(If NO) – Ask the patient about a better time to complete the interview

First thank you again for participating in this project. Do you have any questions before we get started?

(If YES), - answer to the best of my ability

(If NO) - Great!

Now I’ll go over some information about the interview.

This is a research study. The purpose of this study is to understand patient perceptions of immediate postpartum LARC, to help further explain black-white differences in immediate postpartum LARC uptake.

This interview will last about 45-minutes. Everything we discuss is confidential. I will be taking notes and record our conversation to be sure I don’t miss anything you say. I will use my notes and the recording to finish our data analysis. Although we may use some of your comments in the final analysis and write-up, no responses will be attached to any names. Your answers will remain anonymous. But only approved study staff here at UNC will have access to the data.

I will also send you the $25 gift card when we are finished with the interview.

If you have any questions during the interview, please let me know. If we need to change any answers as we move along, we can go back. If we need to stop, we can.

Do you have any questions for me now?

(If YES), - answer to the best of my ability

(If NO) - Great!

If you have questions at any point after we talk today, please contact me. My phone number and email address are available on the consent form.

1. **Organization – Prenatal counseling: We’re going to start by talking a bit about prenatal care, that is the care you received during this pregnancy. Let’s get started.**
   1. Were you able to get prenatal care? – Probe: Did you see a doctor, nurse, or midwife to get care about your health and your baby’s health while you were pregnant)?
      1. If **NO** – What were some of the reasons you did not receive care? (Skip to question 3 after the patient answers)
      2. If **YES** – Go to the question 1.2.
   2. Where did you get care?
      1. Probe – Was it from a doctor’s office, local health department, or other medical facility?
   3. How often were you able to go to your scheduled prenatal care visits?
      1. Probe – Did you go to all of your visits, most of your visits, half of your visits, a few of your visits?
         1. Probe – If they say they were not able to attend all of the scheduled visits, ask: What were some of the reasons you were unable to attend those visits?
2. **Organization –Birth control counseling**

Ok. We’re going to keep talking about your prenatal care, but we’re going to focus a little more on things you talked about with your doctor/nurse/midwife

- 1. When you were going to your prenatal visits, did you talk about birth control after this delivery?
     1. If **NO** – skip to question 3
     2. If **YES** – What kinds of things did you talk about regarding birth control?
  2. When you were going to your prenatal visits what information, if any, did your doctor/nurse/midwife give you about birth control?
     1. If **NONE** – skip to question 3
     2. Probe – Did they give you any recommendations about the type of birth control you should get? Did they give you any advice about when you should get it?
     3. Probe – What did you think about the advice they gave you?
     4. Probe – How often did they talk to you about it?
     5. Probe – What, if anything, do you wish had been different about what they chose to talk about with you?

1. **Health Belief – Birth Control**

During this pregnancy where else did you get information about birth control besides the doctor/nurse/midwife?

- 1. If **YES** Probe – What did you think about the information you received?
     1. Probe – Was the information good? Correct? Useful?
     2. What information was more valuable for you, the information your doctor/nurse/midwife gave you, or the information your (other source of information) gave you?
  2. If **NO** – skip to question 4.

1. **Health Belief – Birth Control**

Now let’s spend some time talking about the decisions you’ve made about the birth control you might use in the future. You might not have made a final decision just yet, but I want to get a sense of the options you are considering

- 1. How do you feel about using birth control?
  2. Are you thinking of using birth control when you leave the hospital?
     1. If No – Skip to question 4.2.3
     2. Probe – What type of birth control, if any, are you thinking of beginning/starting it? When?
     3. Probe – When you were deciding about (name/type of birth control) what kinds of things were you were considering?
        - 1. *Probe* - If the patient names an IUD/Implant – How soon are you planning to get the (IUD/Implant)?

1. **Organization –Birth control counseling**

- 1. Has the doctor/nurse/midwife given you any information about birth control?
     1. What did they say?

1. **Perceived Need – Perceptions of pregnancy**

These next few questions are about pregnancy and planning for pregnancy.

- 1. How soon do you think a woman can get pregnant after having a baby?
  2. I know that you just had a baby, and you might not be thinking about getting pregnant right away or having anymore children, but compared to most women how do soon do you think you could get pregnant again?
     1. **Note –For patients with any long-acting method** – Before getting your (IUD/Implant) how soon do you think you would have been able to get pregnant?

We’re almost done. We have a few more questions about planning for pregnancy

- 1. Do you feel you can decide when to or when not to get pregnant?

1. **Health Beliefs – Pregnancy/Reproductive Planning (5 minutes)**

Tell me what you think about planning for pregnancy.

- 1. Probe – How important is it to plan to pregnancy? How easy is it to plan for pregnancy? What gets in the way of planning for pregnancy??

1. **Is there anything else you want to tell me about birth control or planning for pregnancy?**

Thank you (Ms. patient name). We value your response and appreciate that you participated in this study. Again, we may use some of your comments in our final write-up, but you will not be identified by name. This interview and the data I have will remain confidential. Do you have any questions for me?

(If YES), - answer to the best of my ability

(If NO) – If you think of any later feel free to contact me

(Collect address where to send gift card). Thank you again (Ms. patient name) Have a good day
